# A Novel Method for the Dynamic Coefficients Identification of Journal Bearings Using Kalman Filter

**DOI:** 10.3390/s20020565

**Published:** 2020-01-20

**Authors:** Yang Kang, Zhanqun Shi, Hao Zhang, Dong Zhen, Fengshou Gu

**Affiliations:** 1 Tianjin Key Laboratory of Power Transmission and Safety Technology for New Energy Vehicles, School of Mechanical Engineering, Hebei University of Technology, Tianjin 300130, China; kangyang1125@163.com (Y.K.); z_shi@hebut.edu.cn (Z.S.); d.zhen@hebut.edu.cn (D.Z.); 2 Centre for Efficiency and Performance Engineering, University of Huddersfield, Huddersfield HD1 3DH, UK; f.gu@hud.ac.uk

**Keywords:** journal bearing, dynamic coefficients, identification, displacements, Kalman filter

## Abstract

The dynamic coefficients identification of journal bearings is essential for instability analysis of rotation machinery. Aiming at the measured displacement of a single location, an improvement method associated with the Kalman filter is proposed to estimate the bearing dynamic coefficients. Firstly, a finite element model of the flexible rotor-bearing system was established and then modified by the modal test. Secondly, the model-based identification procedure was derived, in which the displacements of the shaft at bearings locations were estimated by the Kalman filter algorithm to identify the dynamic coefficients. Finally, considering the effect of the different process noise covariance, the corresponding numerical simulations were carried out to validate the preliminary accuracy. Furthermore, experimental tests were conducted to confirm the practicality, where the real stiffness and damping were comprehensively identified under the different operating conditions. The results show that the proposed method is not only highly accurate, but also stable under different measured locations. Compared with the conventional method, this study presents a more than high practicality approach to identify dynamic coefficients, including under the resonance condition. With high efficiency, it can be extended to predict the dynamic behaviour of rotor-bearing systems.

## 1. Introduction

In a rotor-bearing system, the bearing dynamic coefficients affect the dynamic behaviour of the system directly, such as the critical speed, imbalance response, and stability performance. Many investigations based on the theoretical model have been carried out to calculate the dynamic coefficients [1,2,3,4]. However, the simplification in modeling inevitably leads to errors between the calculated and actual values. Thus, a high-accuracy identification method is necessary in terms of the assessment for the overall performance and operating conditions of the rotor system.

Many experimental identification methods have been developed to identify the bearing dynamic coefficients based on the model of the system and the measurement displacement, which are often designed based on the excitation methods, such as dynamic loads [5], impulse [6,7], and the imbalance mass [8]. Among these excitation methods, the imbalance mass, which has high accuracy and does not need extra devices, is more widely used in the identification of the bearing dynamic coefficients [9,10] and imbalance information [11,12]. Additionally, some optimization techniques, such as the equivalent dynamic load reconstruction [13], hybrid evolutionary algorithm [14], and the Bayesian inference [15], are combined with the imbalance excitation methods to improve the accuracy in bearing dynamic coefficients. Moreover, the ill-problems in the identification process are also a concern and should be avoided by combining the dynamic equations from the unbalance response and the proposed complementary equations [16].

For a flexible rotor-bearing system, the accuracy of the model-based identification method of the dynamic coefficients is sensitive to the system model and the displacement of the shaft at bearings. If the finite element model is perfect, and the displacement can be accurately measured, the actual dynamic coefficients can be identified directly. However, in real applications, the displacement of the shaft at bearings is very difficult to measure directly because the measured location cannot be installed at the bearing node in the finite element model. The measured displacement at the bearing housing also cannot be used to identify the bearing dynamic coefficients directly [5,6,11]. Therefore, to obtain the actual displacement of the shaft at bearings, a conventional method has been proposed and widely used, which is based on the linear interpolation to estimate the displacement of the shaft at both bearings using the measured displacements near every bearing location [9,10,17]. However, if the sensors are far away from the locations of bearings and only the displacement of the single location can be measured, the displacement of the shaft at bearing locations cannot be estimated by this method. For this problem, a double-section interpolation-iteration method, in which the initial dynamic coefficients are used to recover the displacement of the shaft at bearing locations to recalculate the dynamic coefficients, has been designed to identify the dynamic coefficients [18]. However, this method demands a large calculation space and easily causes divergent results.

The main challenge of previous model-based identification methods is to estimate the displacement of the shaft at bearings before the identification, especially when only the displacement of the single location can be measured. The Kalman filter is a system dynamic estimation algorithm which produces an estimation of unknown variables using a series of measurements observed over time containing statistical noise and other inaccuracies. This method has been used successfully in the estimation of the critical parameters of the system, such as force [19,20,21,22], structural damage diagnosis [23], inverse heat conduction [24], pore water electrical conductivity [25], and mobile-robot attitude [26] and dynamic state [27,28,29]. Additionally, compared with other algorithms, such as dual Kalman filter [30], join Kalman filter [31], and even recursive least squares (RLS) [32], Kalman filtering is not only easier to achieve for estimating the main parameters in the discrete-time dynamic system, but also can save computing time.

Therefore, in this paper, the Kalman filter is employed in estimating the displacement of the shaft at bearing locations using the measured displacement of the single location. With the estimated displacements and system model, which are transformed and reordered into the frequency domain, the bearing dynamic coefficients can be identified by the least-squares method [33]. Finally, numerical simulations are carried out to validate the accuracy of this method, considering the effect of the process noise covariance. Moreover, some comparisons between the proposed and conventional method are conducted, where the results show that this method has greater practicality and is more stable, including when the system is in the resonance condition.

The structure of this paper is organized as follows. Section 2 describes the experimental test rig and corresponding updated model of the system, which contains the flexible shaft, bearing supporting, and the discs. In Section 3, an identification method is presented. In Section 4, numerical simulations are carried out. Section 5 provides the experimental investigation, in which the imbalance mass is used to excite the flexible system supported by a pair of journal bearings. Section 6 presents the conclusion of the proposed method.

## 2. Test Rig and Rotor Modeling

### 2.1. Test Rig

**Figure 1:**
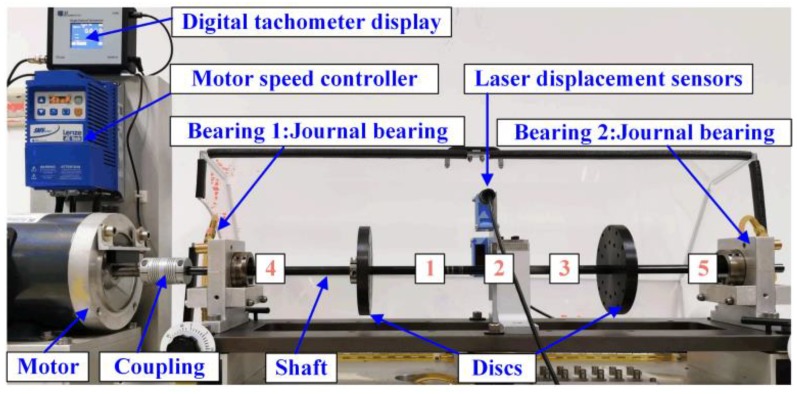
Rotor bearing system.

The rotor-bearing test rig is shown in Figure 1. The shaft was supported by a pair of journal bearings (bearing 1 and bearing 2). Two steel discs were installed symmetrically on the shaft. The laser displacement sensors were used to measure the displacement of shaft in the horizontal and vertical directions, respectively. A three-phase electric motor connected the rotor through a flexible coupling. The rotational speed could be controlled by a variable frequency AC drive. The parameters of the test rig are given in Table 1.

### 2.2. Modeling

**Figure 2:**
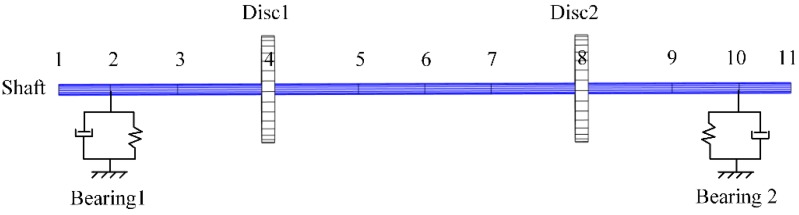
Finite element model.

The corresponding finite element model of the test rig, as shown in Figure 2, was developed. The shaft was modeled by several mass nodes connected with Timoshenko beam elements [34]. Each of the shaft elements had two nodes. Each of the nodes had four degrees of freedom (DOF), including two translational and two rotational. Node 2 and node 10 represent bearing 1 and bearing 2, respectively. Node 4 and node 8 represent the discs’ locations.

The linear equation of motion for the system can be described as:

(1) $$M_{R}\ddot{q}\left(t\right)+\left(\Omega G_{R}+\left(C_{R}+C_{B}\right)\right)\dot{q}\left(t\right)+\left(K_{R}+K_{B}\right)q\left(t\right)=f\left(t\right),$$

where *M_R_*, *C_R_*, *G_R_*, and *K_R_* are the global matrix of lumped masses and inertial, damping, gyroscopic, and stiffness matrices of the rotors, respectively. *K_B_* and *C_B_* are the stiffness and damping matrix, which are required to be identified. Ω is the rotation speed. *f* (*t*) is the imbalance force vector. $\ddot{q}\left(t\right)$, $\dot{q}\left(t\right)$, and *q* (*t*) are vectors of acceleration, velocity, and displacements, respectively, which are written as

(2) $$q={\left[q_{1}\cdots q_{B1}\cdots q_{B2}\cdots q_{n}\right]}^{T},q_{i}=\left[\begin{array}{cccc} x_{i} & y_{i} & \theta_{xi} & \theta_{yi} \end{array}\right],$$

(3) $$f={\left[f_{1}\cdots f_{u}\cdots f_{n}\right]}^{T},i=1-n,$$

where *n* is the number of degrees of freedom, *q_B_*_1_ and *q_B_*_2_ are the displacement of the shaft at bearing 1 and 2 locations, respectively, *x_i_* and *y_i_* are the translational displacements in the horizontal and vertical, respectively, *θ_xi_* and *θ_yi_* are the rotational displacements in the horizontal and vertical, respectively. The imbalance mass was inputted into the model to develop the imbalance force *f_u_*, which is equal to:

(4) $$f_{u}=\left\{\begin{array}{c} f_{x}\\ f_{y}\\ 0\\ 0 \end{array}\right\}=mr\omega^{2}\left\{\begin{array}{c} \cos\left(\omega t+\varphi_{1}\right)\\ \sin\left(\omega t+\varphi_{2}\right)\\ 0\\ 0 \end{array}\right\},$$

where *m* is the imbalance mass, *r* is the distributing radius of the imbalance mass, *ω* = Ω is the imbalance excitation frequency, *φ* is the phase.

**Figure 3:**
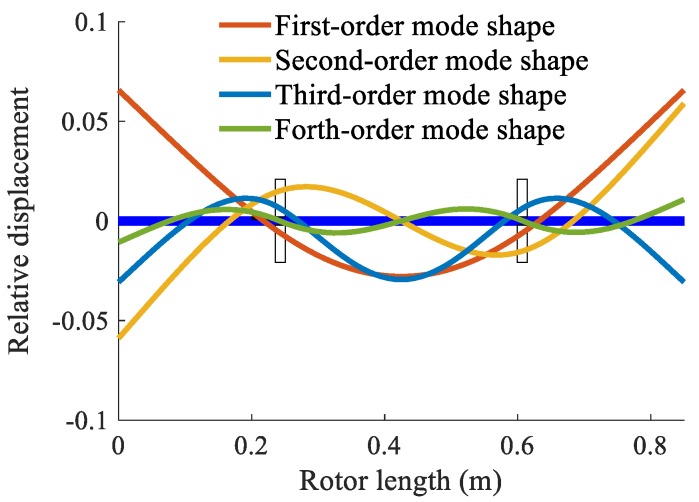
Theoretical mode shapes of the shaft with discs.

The finite element model was then updated to improve its precision. Firstly, the experimental modal parameters and the frequency responses of the shaft with discs were measured. Then, the mass matrix of the model was optimized by the Nelder–Mead simplex optimization algorithm [35]. Finally, the updated model was verified by the comparison of the frequency responses between the calculation and measurement. The theoretical first-four order mode shapes are shown in Figure 3. The modal frequencies obtained from the initial model, the updated model, and measured values are given in Table 2. It can be found that the modal frequencies of the updated finite element model were closer to the measured data than the initial model. Therefore, the finite element model was updated successfully.

## 3. Proposed Method

**Figure 4:**
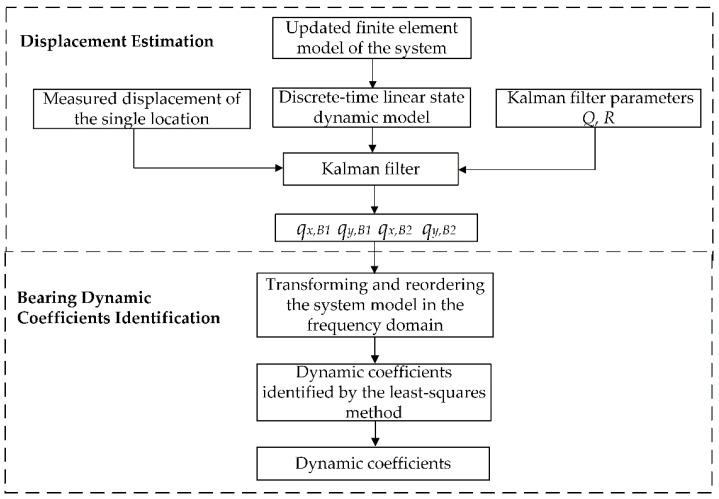
Flowchart of the proposed method.

Most identification methods of the bearing dynamic coefficients are based on the estimated displacement of the shaft at bearing locations. Therefore, an improved identification method based on the Kalman filter is proposed using the measured displacement from a single location to identify the bearing dynamic coefficients. Firstly, applying the system parameters to the model of the system, the actual displacements of both bearings are estimated by Kalman filter using the measured displacement of the single location. Secondly, using the estimated displacement, the actual dynamic coefficients are identified by the least-squares method. The flowchart of the proposed method is given in Figure 4.

### 3.1. Displacement Estimation

Generally, the accuracy of the identification method mainly depends on the displacement of the shaft at bearing locations, because it is very difficult to measure directly. Therefore, in this section, based on the Kalman filter, the displacement of the shaft at bearing locations was estimated using a single measurement location outside of the bearing. To achieve the Kalman filter in high accuracy, the theoretical dynamic coefficients of different rotation speeds, which are close to the actual value and calculated by the perturbation method [36], were applied to the model of the system. 

The Kalman filter requires the state-space representation of the governing equation of the system and Equation (1) should be transformed as:

(5) $$\dot{X}(t)=A_{c}X(t)+B_{c}f(t),$$

(6) $$Y(t)=G_{c}X(t),$$

where $X(t)=\left\{\begin{array}{cccccccc} q_{1} & q_{2} & \cdots & q_{n} & {\dot{q}}_{1} & {\dot{q}}_{2} & \cdots & {\dot{q}}_{n} \end{array}\right\}$ is the state vectors, *A_c_* is the system matrix, *B_c_* is the input matrix, *G_c_* is the output influence matrix, *Y*(*t*) represents the measurement vector.

(7) $$A_{c}=\left[\begin{array}{cc} 0_{n\times n} & I_{n\times n}\\ -M_{R}{}^{-1}(K_{R}+K_{B\text{\_}theoretical}) & -M_{R}{}^{-1}(C_{R}+C_{B\text{\_}theoretical}) \end{array}\right],$$

(8) $$B_{c}=\left[\begin{array}{c} 0_{n\times n}\\ M_{R}{}^{-1} \end{array}\right],$$

(9) $$G_{c}=\left[\begin{array}{cc} I_{n\times n} & 0_{n\times n} \end{array}\right],$$

where *K_B_theoretical_* and *C_B_theoretical_* are the theoretical values of the stiffness and damping matrix, respectively. 

Then, discretize (5) and (6) over the time interval of the ∆*t*, as given in the following,

(10) X(k+1)=AX(k)+Bf(k)+W(k),

(11) Z(k)=GX(k)+V(k),

where *k* is the time index, *X*(*k*) is the state vector, *f*(*k*) is the force vector, *Z*(*k*) denotes the measurement vector, *A* is the state transition matrix, *B* is the input matrix, *G* represents the output influence matrix, and *W*(*k*) and *V*(*k*) are the system and measurement noise vectors, which are assumed to be zero mean and white noise with variance *Q* and *R*, respectively.

Based on the Kalman filter, *q_x,B_*_1_, the displacement of the shaft at bearing 1 in the horizontal direction was estimated by the following three steps.

Step 1: State predicted.

(12) $$\bar{X}\left(k/k-1\right)=A\bar{X}\left(k-1/k-1\right)+Bf\left(k-1\right),$$

(13) $$P\left(k/k-1\right)=AP(k-1/k-1)A^{T}+Q$$

Step 2: Kalman gain calculated.

(14) $$S(k)=G_{x,B1}P(k/k-1)G_{x,B1}{}^{T}+R,$$

(15) $$K_{g}(k)=P\left(k/k-1\right)G_{x,B1}{}^{T}S{\left(K\right)}^{-1},$$

Step 3: State updated.

(16) $$P(k/k)=\left(I-K_{g}(k)G_{x,B1}\right)P\left(k/k-1\right),$$

(17) $$\bar{Z}(k)=Z(k)-G_{x,B1}\bar{X}(k/k-1),$$

(18) $$\bar{X}\left(k/k\right)=\bar{X}\left(k/k-1\right)+K_{g}(k)\bar{Z}(k),$$

where *G_x, B_*_1_ is the output influence matrix corresponding to the bearing 1 node at the horizontal direction and can be obtained from *G*, $\bar{X}\left(k/k-1\right)$ is the predicted state vectors, $\bar{X}\left(k/k\right)$ denotes the updated state, which is the estimated displacement of *q_x, B_*_1_, *P*(*k/k*−1) and *P*(*k/k*) represent the predicted and updated covariance matrix, respectively, *S*(*k*) is the innovation covariance matrix, *K_g_* is the Kalman gain, *Z*(*k*) represents the innovation matrix.

After the above calculation, the displacement of the shaft at bearing 1 location in the horizontal direction (*q_x, B_*_1_) has been obtained. Additionally, the displacement of the shaft at bearing 1 in the vertical direction (*q_y, B_*_1_)_,_ and bearing 2 in both directions (*q_x, B_*_2_, *q_y, B_*_2_) can be estimated by the same procedure.

### 3.2. Bearing Dynamic Coefficients Identification

The displacements of the shaft at both bearing locations can be determined, as shown in the last section. With the estimated displacement, the bearing dynamic coefficients can be identified in the following process.

In this study, the identification method was designed with the system model in the frequency domain. After applying the Fourier transform, Equation (1) can be expressed as:

(19) $$\left[K_{R}+K_{B}-\Omega^{2}M_{R}+i\Omega \left(C_{R}+C_{B}\right)-i\Omega^{2}G_{R}\right]q_{u}=F,$$

and the transfer function between imbalance extrication and displacement is:

(20) $$H=\left[K_{R}+K_{B}-\Omega^{2}M_{R}+i\Omega \left(C_{R}+C_{B}\right)-i\Omega^{2}G_{R}\right]=H_{R\left(\Omega \right)}+H_{B\left(\Omega \right)},$$

With:

(21) $$H_{R\left(\Omega \right)}=\left[K_{R}-\Omega^{2}M_{R}+i\Omega C_{R}-i\Omega^{2}G_{R}\right],$$

(22) $$H_{B\left(\Omega \right)}=\left[K_{B}+i\Omega C_{B}\right]$$

Thus, the equation of the motion of the rotor-bearing system can be represented by *H_R(_*_Ω*)*_ and *H_B_*_(Ω)_ and rewritten as below:

(23) $$\left(H_{R(\Omega )}+H_{B(\Omega )}\right)q_{u}=F,$$

where *q_u_* is the displacement vectors of the system, which is in the complex form and caused by the imbalance force. 

To identify the dynamic coefficients directly, the algebraic system of Equation (23) was reordered by the use of matrix operation to bring the estimated displacements of the shaft at both bearing locations into the upper rows. The displacement vector can be rewritten as:

(24) $${\bar{q}}_{u}={\left[\begin{array}{ccc} z_{B1} & z_{B2} & z_{u} \end{array}\right]}^{T},$$

where the vector *z_u_* represents the vector of unknown rotor displacements. *z_B_*_1_ and *z_B_*_2_ represent the estimated displacement vectors of the shaft at bearing locations, respectively, as:

(25) $$z_{B1}={\left[q_{x,B1},q_{y,B1}\right]}^{T},$$

(26) $$z_{B2}={\left[q_{x,B2},q_{y,B2}\right]}^{T}.$$

The system of Equation (23) can be rewritten and shown as:

(27) $${\bar{H}}_{R}\left\{\begin{array}{c} z_{B1}\\ z_{B2}\\ z_{u} \end{array}\right\}+\left[\begin{array}{ccc} H_{B1} & 0 & 0\\ 0 & H_{B2} & 0\\ 0 & 0 & 0 \end{array}\right]\left\{\begin{array}{c} z_{B1}\\ z_{B2}\\ z_{u} \end{array}\right\}=\left\{\begin{array}{c} 0\\ 0\\ \bar{F} \end{array}\right\},$$

With:

(28) $$H_{b}=\left[\begin{array}{cc} K_{xx,b}+j\Omega C_{xx,b} & K_{xy,b}+j\Omega C_{xy,b}\\ K_{yx,b}+j\Omega C_{yx,b} & K_{yy,b}+j\Omega C_{yy,b} \end{array}\right],b=B1,B2.$$

In order to calculate conveniently, ${\bar{H}}_{R}$ is partitioned into a sub-matrix like below:

(29) $$\left[\begin{array}{ccc} {\bar{H}}_{R11} & {\bar{H}}_{R12} & {\bar{H}}_{R13}\\ {\bar{H}}_{R21} & {\bar{H}}_{R22} & {\bar{H}}_{R23}\\ {\bar{H}}_{R31} & {\bar{H}}_{R32} & {\bar{H}}_{R33} \end{array}\right]\left\{\begin{array}{c} z_{B1}\\ z_{B2}\\ z_{u} \end{array}\right\}=\left\{\begin{array}{c} -H_{B1}z_{B1}\\ -H_{B2}z_{B2}\\ \bar{F} \end{array}\right\},$$

where the displacement of the shaft at bearing locations and the imbalance force vector can be measured and calculated directly. Thus, the third row of Equation (29), *z_u_* can be expressed as:

(30) $$z_{u}={\bar{H}}_{R33}^{-1}\left\{\bar{F}-{\bar{H}}_{R31}z_{B1}-{\bar{H}}_{R32}z_{B2}\right\}.$$

Then, from the first and second row of Equation (29), the following equations can be obtained:

(31) $$H_{B1}z_{B1}=-{\bar{H}}_{R11}z_{B1}-{\bar{H}}_{R12}z_{B2}-{\bar{H}}_{R13}z_{u}=f_{B1},$$

(32) $$H_{B2}z_{B2}=-{\bar{H}}_{R21}z_{B2}-{\bar{H}}_{R22}z_{B2}-{\bar{H}}_{R23}z_{u}=f_{B2}.$$

Since the *z_u_* can be calculated from Equation (30) and the *f_B_*_1_ and *f_B_*_2_ are known, the bearing dynamic coefficients can be identified by:

(33) [E(Ω)]{β(Ω)}={D(Ω)},

where *E* is the displacement matrix of both bearings, *β* is the parameters matrix of the bearing dynamic coefficients, which are required to be identified, and *D* is the system matrix that contains *f_B_*_1_ and *f_B_*_2_.

(34) $$\begin{array}{l} E=[\begin{array}{cccccccc} q_{x,B1} & q_{y,B1} & 0 & 0 & 0 & 0 & 0 & 0\\ 0 & 0 & q_{x,B1} & q_{y,B1} & 0 & 0 & 0 & 0\\ 0 & 0 & 0 & 0 & q_{x,B2} & q_{y,B2} & 0 & 0\\ 0 & 0 & 0 & 0 & 0 & 0 & q_{x,B2} & q_{y,B2} \end{array}\\ \begin{array}{cccccccc} j\Omega q_{x,B1} & j\Omega q_{y,B1} & 0 & 0 & 0 & 0 & 0 & 0\\ 0 & 0 & j\Omega q_{x,B1} & j\Omega q_{y,B1} & 0 & 0 & 0 & 0\\ 0 & 0 & 0 & 0 & j\Omega q_{x,B2} & j\Omega q_{y,B2} & 0 & 0\\ 0 & 0 & 0 & 0 & 0 & 0 & j\Omega q_{x,B2} & j\Omega q_{y,B2} \end{array}] \end{array},$$

(35) $$\left\{D\left(\Omega \right)\right\}={\left\{\begin{array}{cccc} f_{B1}\left(1\right) & f_{B1}\left(2\right) & f_{B2}\left(1\right) & f_{B2}\left(2\right) \end{array}\right\}}^{T},$$

(36) {β(Ω)}16×1={Kxx,B1Kxy,B1Kyx,B1Kyy,B1Kxx,B2Kxy,B2Kyx,B2Kyy,B2 Cxx,B1Cxy,B1Cyx,B1Cyy,B1Cxx,B2Cxy,B2Cyx,B2Cyy,B2}T

Finally, the bearing dynamic coefficients can be calculated by the least-squares method in one operation, as:

(37) $$\left\{\beta \left(\Omega \right)\right\}={\left({\left[A\left(\Omega \right)\right]}^{T}\left[A\left(\Omega \right)\right]\right)}^{-1}{\left[A\left(\Omega \right)\right]}^{T}\left\{D\left(\Omega \right)\right\}$$

## 4. Simulations

In this section, to validate the accuracy of the proposed method, the numerical simulations are conducted, where the effect of the variance Q for the bearing dynamic coefficients is considered and discussed. 

Firstly, the imbalance mass was added to generate the corresponding imbalance force shown in Equation (4). The assumed dynamic coefficients of both bearings at rotation speeds from 600 to 4800 rpm were added to the model to generate the simulation displacement and achieve the Kalman filter, in which the dynamic coefficients of both bearings were the same value. During the range of the rotation speeds, the displacement of each node could be obtained by the simulation calculation. Secondly, based on the Kalman filter, the displacements of shafts at the bearing locations were estimated by the displacement of node 6, where the estimated displacements were validated by the actual value of the bearing nodes 2 and 10. Finally, with the estimated displacement, the stiffness and damping coefficients of both bearings were identified and compared with the assumed values. The details of the imbalance mass are given in Table 3.

**Figure 5:**
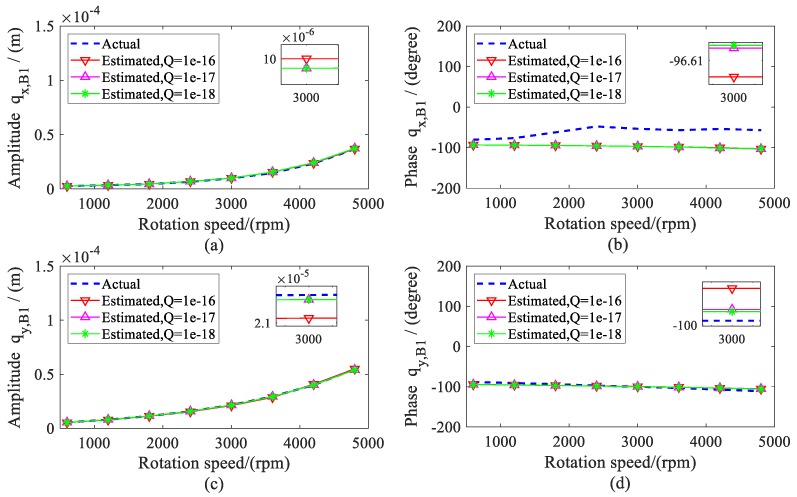
Actual and estimated displacements of bearing 1; (**a**) amplitude of *q_x, B_*_1_; (**b**) phase of *q_x,B_*_1_; (**c**) Amplitude of *q_y, B_*_1_; (**d**) phase of *q_y, B_*_1_.

**Figure 6:**
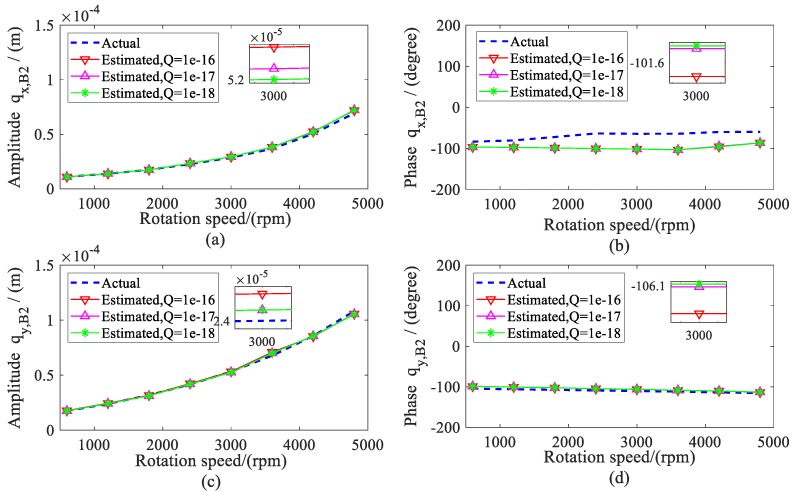
Actual and estimated displacements of bearing 2. (**a**) amplitude of *q_x, B_*_2_; (**b**) phase of *q_x,B_*_2_; (**c**) amplitude of *q_y, B_*_2_; (**d**) phase of *q_y, B_*_2_.

The amplitude and phase of the estimated displacement during the range of the rotation speeds are shown in Figure 5 and Figure 6. The amplitude of bearing 2 was larger than the bearing 1 because the imbalance mass was added to disc 2 (node 8), which was close to bearing 2. To validate the estimation results, the actual displacements of shafts at the bearing locations (node 2 and 10) were also given in the following. It can be found that the estimated values matched quite well with the actual value, especially when the variance *Q* was less than 1 × 10^−16^. 

**Figure 7:**
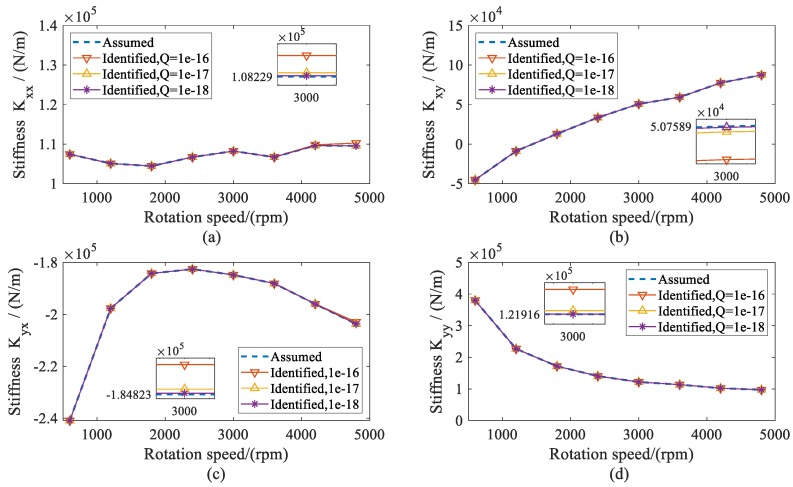
Assumed and identified stiffness coefficients of bearing 2 versus rotation speed; (**a**) direct stiffness coefficient *K_xx_*; (**b**) cross-coupled stiffness coefficient *K_xy_*; (**c**) cross-coupled stiffness coefficients *K_yx_*; (**d**) direct stiffness coefficients *K_yy_*.

**Figure 8:**
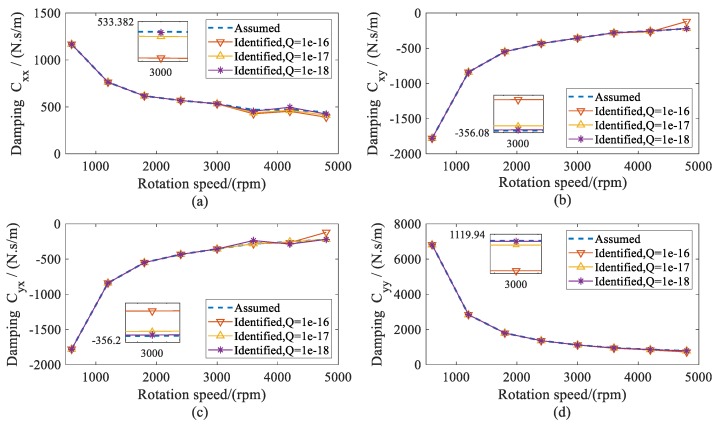
Assumed and identified damping coefficients of bearing 2 versus rotation speed. (**a**) direct damping coefficients *C_xx_*; (**b**) cross-coupled damping coefficients *C_xy_*; (**c**) cross-coupled damping coefficients *C_yx_*; (**d**) direct damping coefficients *C_yy_*.

Using the estimated displacement of shafts at the bearing locations, the dynamic coefficients of both bearings were identified during the range of the rotation speeds. The identified stiffness and damping coefficients of both bearings are presented as the same results. Therefore, the dynamic coefficients of bearing 2 are given in Figure 7 and Figure 8. From the results, it can be seen that all of the identified dynamic coefficients under different variances *Q* agreed well with the assumed coefficients, especially when the variance *Q* was 1 × 10^−18^. Therefore, it can be concluded that, theoretically, the presented method has high confidence in the identification of dynamic coefficients.

## 5. Experimental Analysis

The accuracy of the proposed method was preliminarily validated in the last section. In this section, based on the flexible test rig shown in Figure 2, the identification procedure used the measured displacement for the three cases of locations to determine the stiffness and damping coefficients of a pair of journal bearings. 

### 5.1. Experimental Description

**Figure 9:**
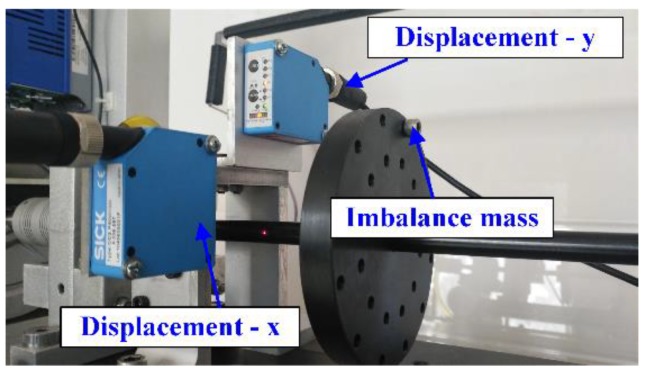
The detail of the displacement sensor.

The imbalance masses as an excitation force were added to disc 1 and disc 2. The laser displacement sensors were used to measure the displacement of the shaft. In this paper, at the range of the rotation speed from 600 rpm to 2200 rpm, the displacement of the three different locations, 1, 2, and 3, were measured and used to identify the stiffness and damping coefficients. The details of the displacement sensors are shown in Figure 9. The parameters of the imbalance mass are given in Table 4. 

To validate the accuracy and practicality of the proposed method, the displacements of the bearing locations and dynamic coefficients were also identified based on the conventional method [17], which requires the measured displacements of the two locations near the bearings. The conventional method is widely used but is not suitable for complex structures because it is sensitive to the selection of the measured locations. The comparisons between the proposed and conventional method are given in Section 5.2.

### 5.2. Results and Discussions

**Figure 10:**
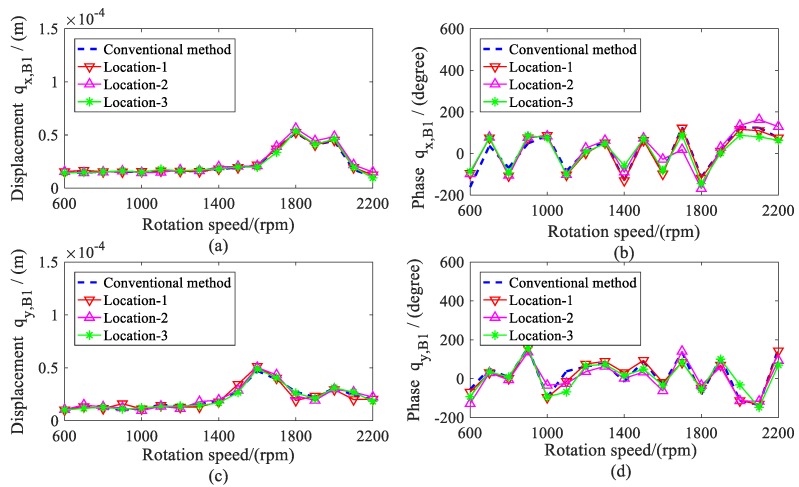
Comparison for displacements of bearing 1. (**a**) amplitude of *q_x, B_*_1_; (**b**) phase of *q_x,B_*_1_; (**c**) amplitude of *q_y, B_*_1_; (**d**) phase of *q_y, B_*_1_.

**Figure 11:**
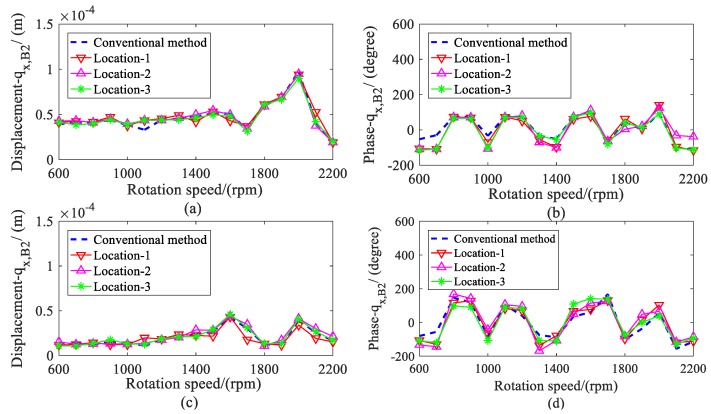
Comparison for displacements of bearing 2. (**a**) amplitude of *q_x, B_*_2_; (**b**) phase of *q_x, B_*_2_; (**c**) amplitude of *q_y, B_*_2_; (**d**) phase of *q_y, B_*_2_.

The amplitude and phase of the shaft displacement at both bearing locations were estimated and are provided in Figure 10 and Figure 11. The amplitude had an evident increase from 1600 to 1900 rpm because of the first-order critical speed. Over the whole range of the rotation speed, it can be seen that the estimated amplitude and phase under different measured displacements agreed well with the conventional method, including at the first-order critical speed.

**Figure 12:**
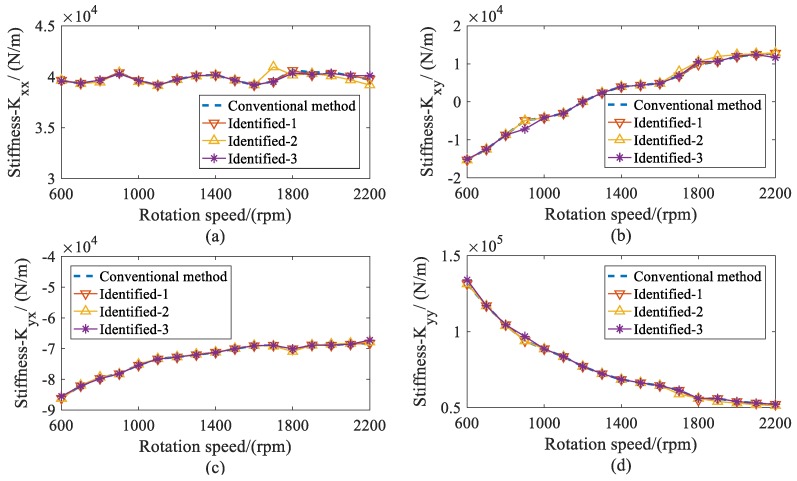
Comparison for stiffness coefficients of bearing 1 versus rotation speed. (**a**) direct stiffness coefficient *K_xx_*; (**b**) cross-coupled stiffness coefficient *K_xy_*; (**c**) cross-coupled stiffness coefficients *K_yx_*; (**d**) direct stiffness coefficients *K_yy_*.

**Figure 13:**
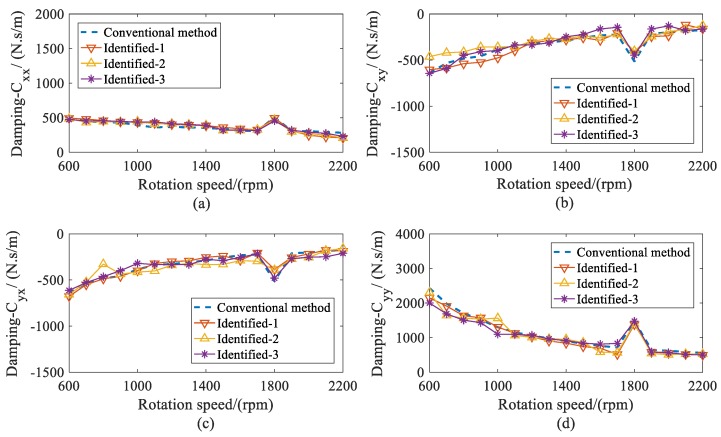
Comparison for damping coefficients of bearing 1 versus rotor speed. (**a**) direct damping coefficients *C_xx_*; (**b**) cross-coupled damping coefficients *C_xy_*; (**c**) cross-coupled damping coefficients *C_yx_*; (**d**) direct damping coefficients *C_yy_*.

With the estimated displacement of bearings, the stiffness and damping coefficients of both bearings were identified. The dynamic coefficients of bearing 1 are shown in Figure 12 and Figure 13. As the rotation speeds increased, the direct stiffness coefficient *K_yy_* had significant decreases, whereas *K_xx_* remained almost constant. The cross-coupled stiffness coefficients *K_xy_* and *K_yx_* were negative and had slight increases over the speed range considered. Besides this, the direct damping coefficients *C_xx_* remained nearly invariant. The cross-coupled damping coefficients (*C_xy_*, *C_yx_*) were similar and smaller than the direct damping coefficients *C_yy_*, which decreased throughout the speed range. From the results, it can be seen that this method not only has a similar accuracy to the conventional method, but is also stable under different measured locations. Compared with the conventional method, this method requiring fewer measured locations makes it more practical for complex structures.

**Figure 14:**
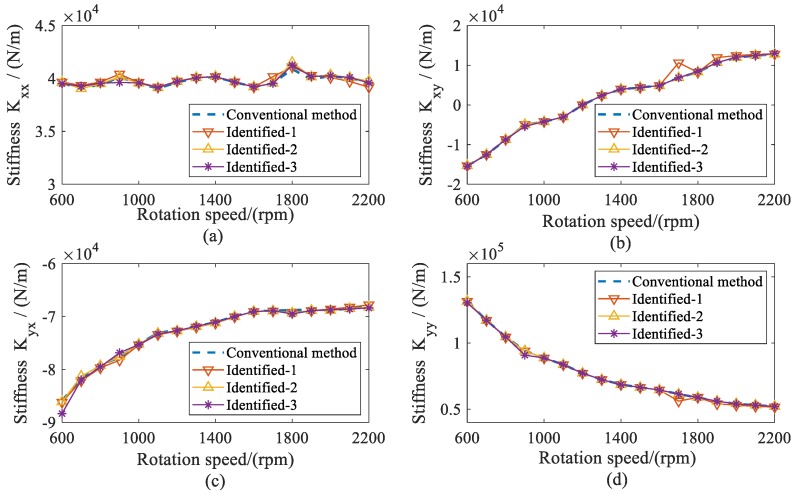
Comparison for stiffness coefficients of bearing 2 versus rotation speed. (**a**) direct stiffness coefficient *K_xx_*; (**b**) cross-coupled stiffness coefficient *K_xy_*; (**c**) cross-coupled stiffness coefficients *K_yx_*; (**d**) direct stiffness coefficients *K_yy_*.

**Figure 15:**
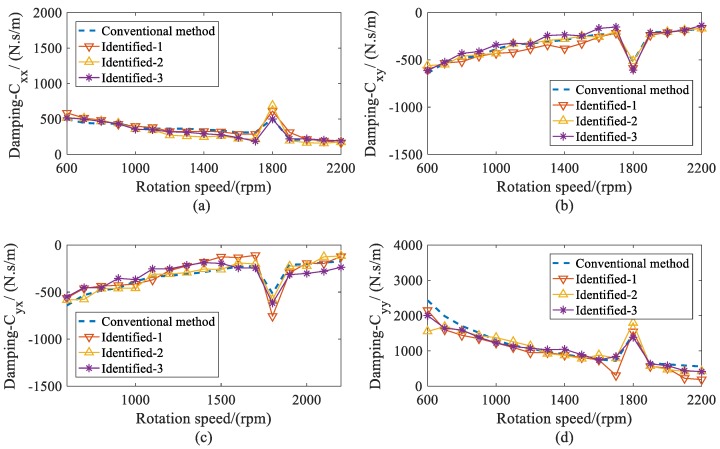
Comparison for damping coefficients of bearing 2 versus rotor speed; (**a**) direct damping coefficients *C_xx_*; (**b**) cross-coupled damping coefficients *C_xy_*; (**c**) cross-coupled damping coefficients *C_yx_*; (**d**) direct damping coefficients *C_yy_*.

In Figure 14 and Figure 15, the identified stiffness and damping coefficients of bearing 2 are provided. Since the machine was a symmetry structure supported by two same journal bearings, the dynamic coefficients of both bearings were similar.

From the comparisons, it can be seen that the identified dynamic coefficients of both bearings obtained satisfying accuracy, including under the resonance condition. Additionally, the proposed method was more flexible in the section of the measured locations because it is stable under different measured locations. Therefore, this method can improve the practicality for the identification of dynamic coefficients in complex structures.

To investigate the influencing features of the measured locations, the errors between the proposed and conventional method were determined by:

(38) $$Error(\%)=\frac{\sum_{i=1}^{N}\left|\frac{\begin{array}{c} Actual \end{array}dynamic\begin{array}{c} coefficients\begin{array}{c} (\Omega )-\begin{array}{c} Estimated \end{array}dynamic\begin{array}{c} coefficients\begin{array}{c} \left(\Omega \right) \end{array} \end{array} \end{array} \end{array}}{\begin{array}{c} Actual \end{array}dynamic\begin{array}{c} coefficients\begin{array}{c} \left(\Omega \right) \end{array} \end{array}}\right|\times 100}{N}.$$

**Figure 16:**
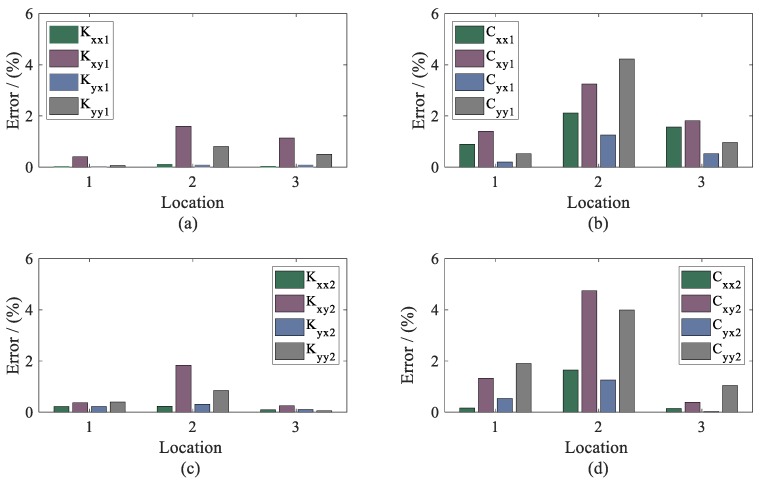
Error between the proposed and conventional method. (**a**) stiffness coefficients of bearing 1; (**b**) damping coefficients of bearing 1; (**c**) stiffness coefficients of bearing 2; (**d**) damping coefficients of bearing 2.

Therefore, the errors of dynamic coefficients under different measured locations were calculated and are presented in Figure 16. From the results, it can be seen that the accuracy of the identification was not affected by the position of the sensor location. Compared with the conventional method, it can be believed that the proposed method, which only requires the displacement of the single location, is more practical and stable, especially in stiffness identification.

In the process of experimental identification, the sampling frequency was 10,240 Hz., while the number of data points was 20,480. The computing time was around 4.402008 s, including estimating the displacement and dynamic coefficients of both bearings. According to the identification results, the proposed method can be carried out for identification in real-time.

## 6. Conclusions

In this paper, the identification of dynamic coefficients was comprehensively investigated in a flexible rotor-journal bearing system. An improvement method was proposed to accurately estimate bearing dynamic coefficients from the measured displacement of a single location.

The procedure of the identification based on the updated finite element model was presented, where the Kalman filter was applied to estimate the actual displacement at the shaft of both bearing locations using the measured displacement from a single location. To validate the effectiveness and accuracy, numerical simulations under different process noise variances were carried out. The results show that all of the identified coefficients matched quite well with the assumed coefficients used for generating the simulation displacements. Furthermore, with the proposed method, experimental tests were also undertaken to identify the dynamic coefficients, including the stiffness and damping parameters. The high precision of the identified coefficients, particularly the stiffness, was confirmed under the operating conditions. Compared with the conventional method, this method is not only stable, including in the resonance condition, but more practical for complex systems. Additionally, the proposed method can achieve the identification of dynamic coefficients in real-time.

It can be concluded that this study can serve as a more efficient and reliable tool for monitoring the stable work of the rotor-bearing system, together with the fault diagnosis.

## Figures and Tables

**Table 1:** The parameters of the test rig.

Property	Value
Rotor Shaft diameter, R	0.01265 m
Maximum rotation speed, Ω	6000 rpm
Mass, m	0.86 kg
Length of rotor, L	0.85 m
Density	7800 kg/m^3^
Rigid discs Inner diameter	0.01265 m
Outer diameter	0.08 m
Thickness	0.015 m
Bearing Diameter	0.01265 m
Length to diameter ratio	1
Radical clearance of bearings	5 × 10^−5^ m

**Table 2:** Measured and theoretical (initial, updated) modal frequencies.

Order	Initial Model (Hz)	Measured (Hz)	Updated Model (Hz)
1	69.26	62.86	62.86
2	129.75	110.20	110.55
3	287.31	279.14	279.14
4	378.53	361.27	362.99

**Table 3:** The parameters of the imbalance mass.

Property	Mass (kg)	Radius (m)	Phase (°)
Node 8	$m_{u2}$ = 0.004	r_2_ = 0.063	90

**Table 4:** The parameters of the imbalance mass.

Property	Mass (kg)	Radius (m)	Phase (°)
Node 4	$m_{u1}$ = 0.0025	r_1_ = 0.063	0
Node 8	$m_{u2}$ = 0.0050	r_2_ = 0.063	90
